# Supplementary material for: Nanoparticles in 472 Human Cerebrospinal Fluid: Changes in Extracellular Vesicle Concentration and miR-21 Expression as a Biomarker for Leptomeningeal Metastasis
Source: Cancers (Basel). 2020 Sep 24;12(10):2745. doi: 10.3390/cancers12102745 (PMC7598615; doi:10.3390/cancers12102745)
Supplement: Supplementary file 1 [file cancers-12-02745-s001.pdf]

Supplementary Materials

Nanoparticles in 472 Human Cerebrospinal Fluid: Changes in Extracellular Vesicle Concentration and miR-21 Expression as a Biomarker for Leptomeningeal metastasis

Kyue-Yim Lee, Ji Hye Im, Weiwei Lin, Ho-Shin Gwak, Jong Heon Kim, Byong Chul Yoo, Tae Hoon Kim, Jong Bae Park, Hyeon Jin Park, Ho-Jin Kim, Ji-Woong Kwon, Sang Hoon Shi, Heon Yoo and Changjin Lee

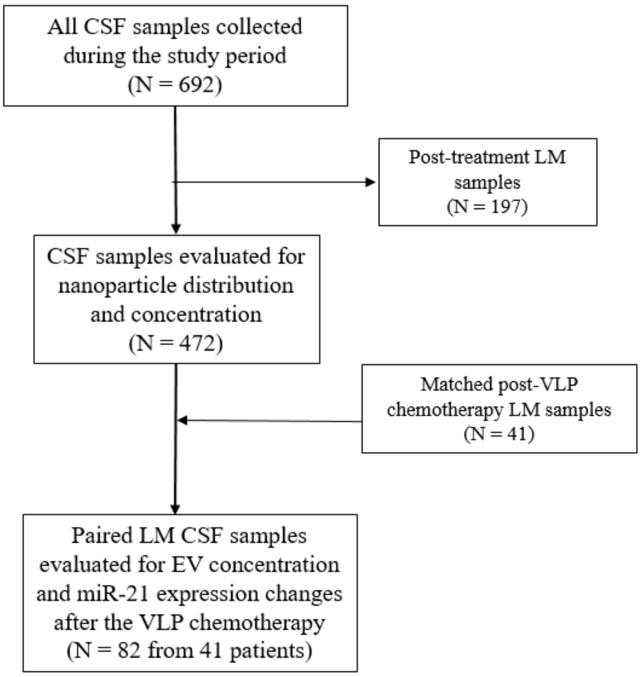

Figure S1. Flow diagram of CSF sample selection. Abbreviations: CSF, cerebrospinal fluid; EV, extracellular vesicles; LM, leptomeningeal metastasis; VLP, ventriculo-lumbar perfusion.

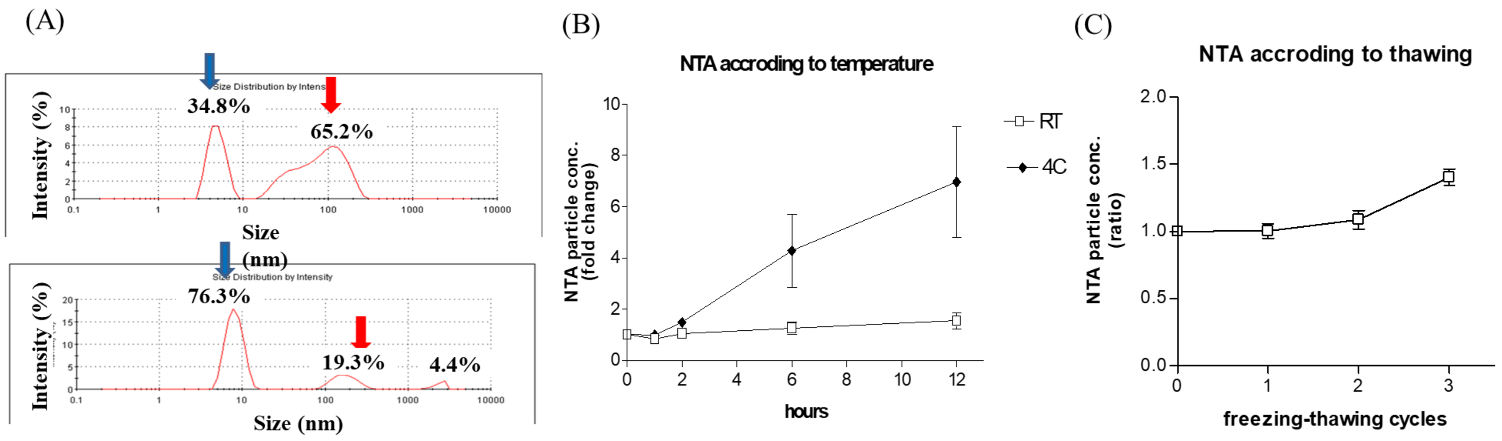

Figure S2. Preliminary analysis of extracellular nanoparticles in CSF. (**A**) DLS-measured change of peaks at nano-range in cerebrospinal fluid of pre-treatment (upper) and post-treatment (lower) from a patient who received wholebrain radiation therapy. (**B**) NTA-measured nanoparticle concentration changes according to different storage temperature before cell-down, and (**C**) freezing-thawing cycles. Error bars represent standard error means of triplicates.

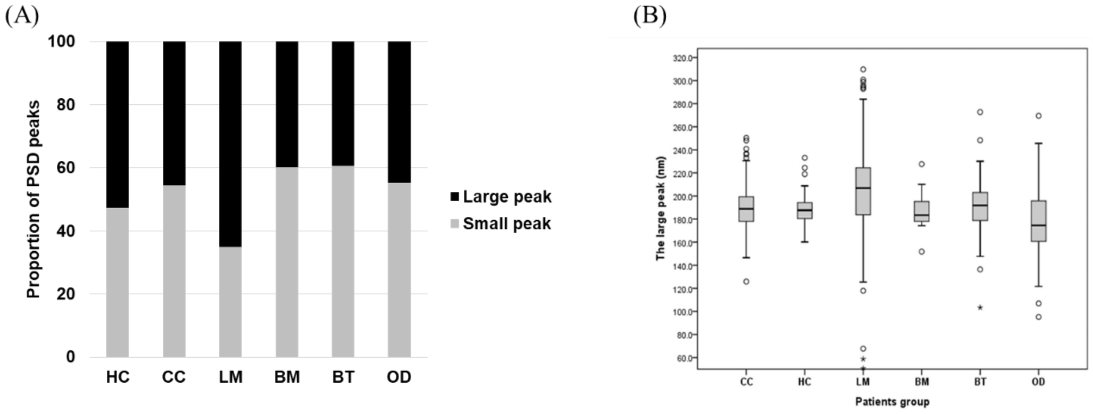

Figure S3. The different distribution of intensity peaks in Dynamic Light Scattering (DLS) analysis. (**A**) The proportion of observed two peaks according to patients' group. Leptomeningeal metastasis (LM) showed significantly lower small peak proportion and higher large peak proportion than other groups (*Student t-test*, $p < 0.001$). (**B**) Box plots representing the size of large peak according to patients' group. LM groups showed significantly larger size than other groups. Thick line is mean value and the box range represents quartile value (*Student t-test*, $p < 0.001$) (CC, cancer control; HC, healthy control; LM, leptomeningeal metastasis; BM, brain metastasis; BT, brain tumor. OD, other disease).

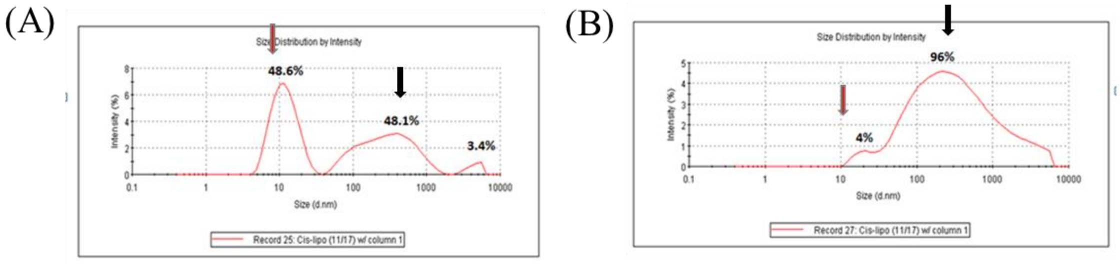

Figure S4. Evaluation of components of nano-range peaks measured by Dynamic Light Scattering (DLS) using Zetasizer Nano system. (**A**) Untreated human CSF shows two peaks as described in the text. (**B**) The small peak was abolished with exosome purification/nucleic acid elimination kit (Exospin®) while the large peak remains. To separate EVs from proteins, nucleic acids, and precipitating agents, the exosome suspension was fractionated using Exo-spin midi-columns (Cell Guidance Systems, St. Louis, MO) according to the manufacturer's instructions. Briefly, CSF was incubated with 500 µl Buffer A for 1 h at room temperature and then centrifuged for 1 h at 16,000 g. The exosome-containing pellet was suspended in 100 µl PBS. The exosome suspension was placed in the top of the column, and the eluate was discarded. The exosome-containing fraction was obtained in 200 µl PBS, and proportion changes were evaluated using Zetasizer Nano analysis.

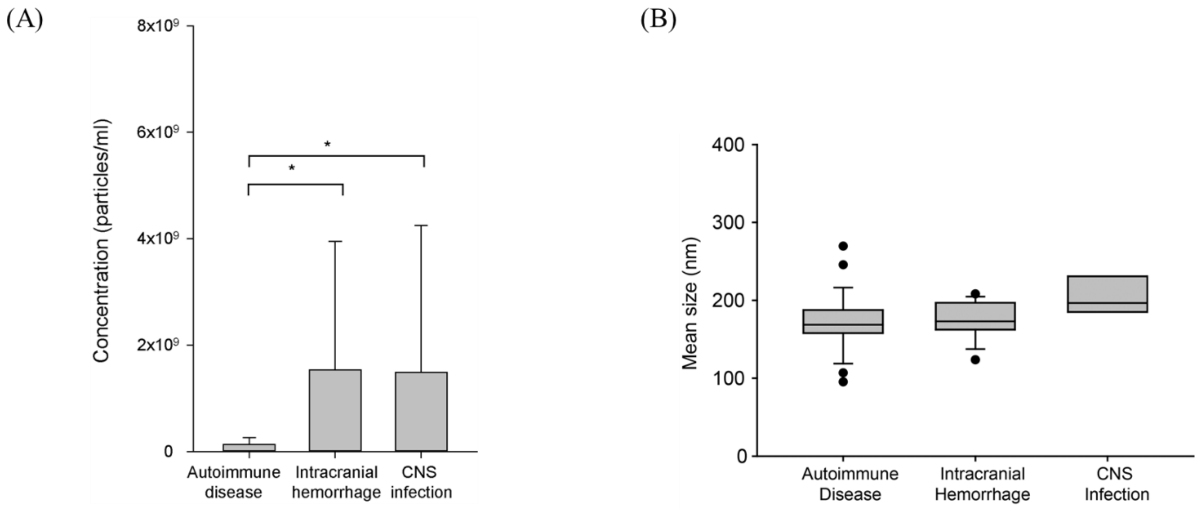

Figure S5. Different (**A**) concentration and (**B**) size of EV in other CNS disease group according to disease entity of autoimmune disease, intracranial hemorrhage and CNS infection (ANOVA, *Dunn's Multiple Comparison*,*$p < 0.05$).

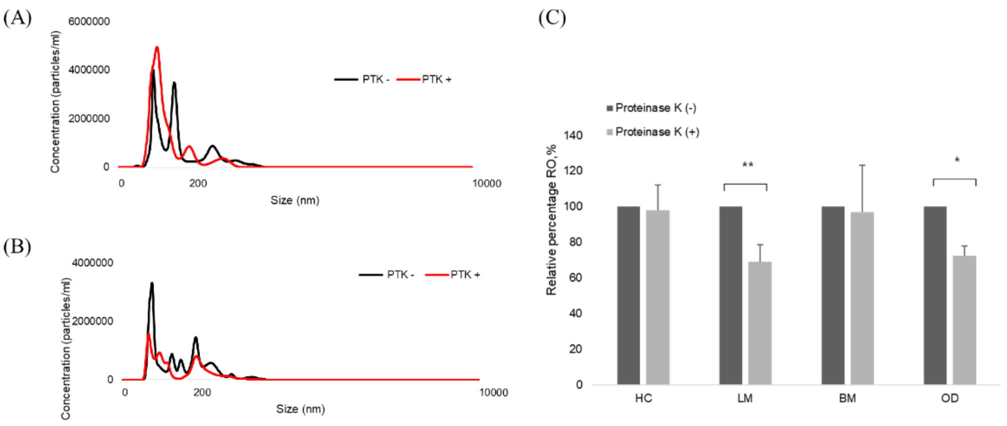

Figure S6. Different patterns of NTA-measured particles before (black line) and after (red line) proteinase treatment. (**A**) Shift of larger particles (>200 nm) with decreased NTA particles, and (**B**) Shift of larger particles (>200 nm) with increased NTA particles. (**C**) Relative ratio of NTA particles according to different CNS disease status. The LM group showed significant higher proportion of non-vesicular, proteinase vulnerable particles compared with other groups (*paired t-test*, $^{**}p < 0.01$, $^{*}p < 0.05$). PTK, proteinase K; HC, healthy control; LM, leptomeningeal metastasis; BM, brain metastasis; OD, other disease.

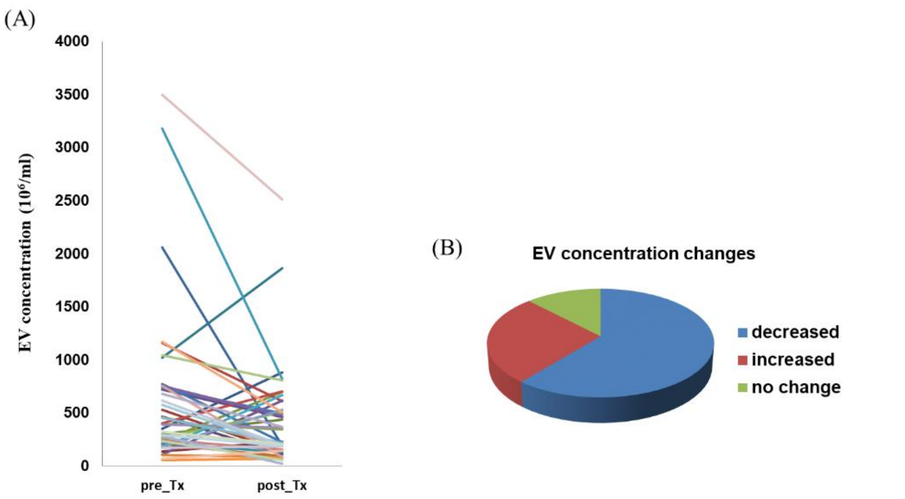

Figure S7. Illustration of extracellular vesicle (EV) concentration change after intra-cerebrospinal fluid chemotherapy. (**A**) Matched pre-treatment and post-treatment exosome concentration of individual patients ($n = 41$), (**B**) Proportion of exosome concentration change of increase, no change (<±20%) and decrease after the treatment.

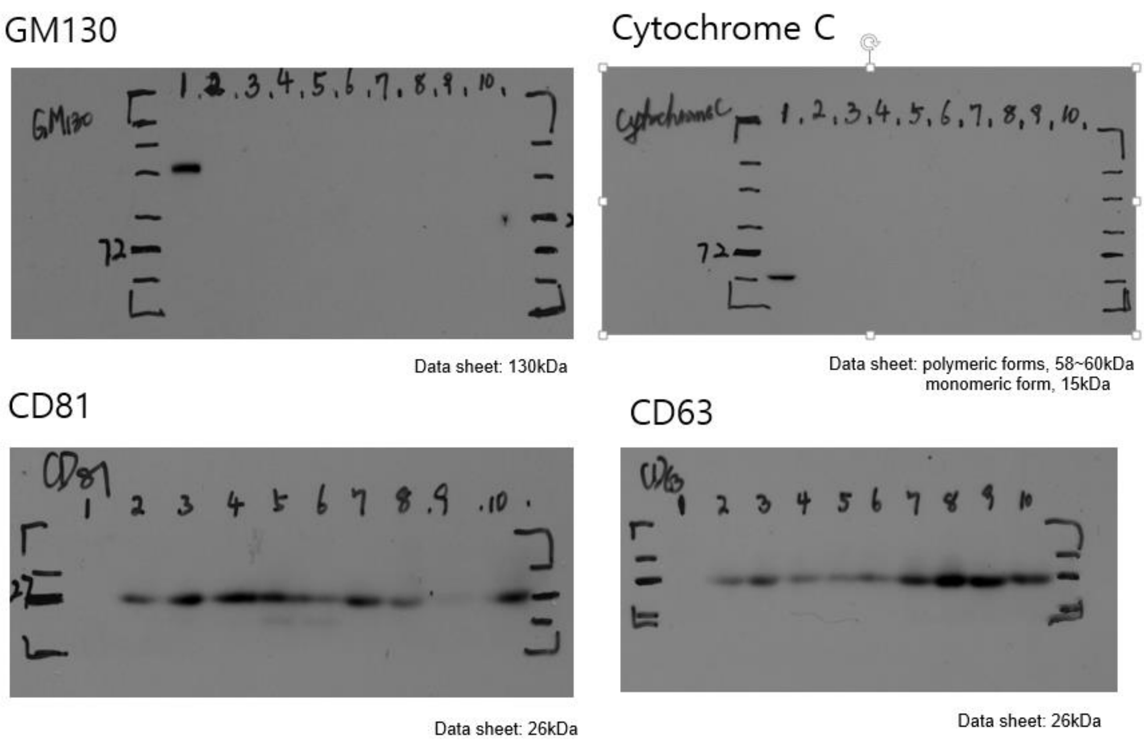

Figure S8. Uncropped Western blots.

Table S1. Comparison of the size of nanoparticles in human CSF between DLS and NTA according to patient groups.

Patient Groups	Cancer Control	Healthy Control	LM	Brain Metastasis	Brain Tumor	Other Disease
[a]DLS Mean diameter ± standard deviation	169 nm (± 98)	106 nm (± 61)	252 nm (± 222)	245 nm (± 262)	164 nm (± 235)	97 nm (± 61)
[b]NTA Mean diameter ± standard error	192 nm (± 22)	189 nm (± 13)	201 nm (± 46)	190 nm (± 19)	191 nm (± 25)	177 nm (± 33)

a: The values of DLS is a mean of the large peak (See details in Methods); b: Samples were measured 3 times and the average values were used for the statistical analysis. Abbreviations: DLS, Dynamic light scattering; LM, Leptomeningeal metastasis; NTA, Nanoparticle tracking analysis.

Table S2. Raw data of miR-21 droplet digital PCR (ddPCR) results in a spread sheet form according to EV concentration changes after the intraventricular chemotherapy in patients with leptomeningeal carcinomatosis from non-small cell lung cancer.

EV change	Pre-Treatment			Post-Treatment			Fold Change of miR-21		
	ddPCR counts	RNA amount (ng) (A260/280, A260/230)	NTA concentration (particles/ml)	ddPCR counts	RNA amount (ng) (A260/280, A260/230)	NTA concentration (particles/ml)	ddPCR counts	RNA normalized	EVs normalized
Increased	12488	285 (1.68/0.03)	4.00×10^8	2292	221 (1.77/0.03)	7.04×10^8	0.18	0.24	0.10
	416	198 (1.15/0.05)	6.51×10^7	996	200 (1.55/0.08)	1.41×10^8	2.39	2.37	1.11
	4949	308 (1.61/0.07)	2.33×10^8	561	358 (1.80/0.04)	6.97×10^8	0.11	0.10	0.04
	2783	360 (1.86/0.03)	3.50×10^8	460	4194 (1.61/0.07)	8.78×10^8	0.17	0.01	0.07
	11594	309 (1.93/1.89)	1.84×10^8	10872	150 (1.60/0.03)	5.30×10^8	0.94	1.93	0.33
No change	1169	555 (1.85/0.03)	3.92×10^8	971	423 (2.02/0.03)	3.59×10^8	0.83	1.09	0.91
	957	795 (1.66/0.09)	5.69×10^7	7	399 (1.85/0.02)	6.57×10^7	0.01	0.01	0.01
	1358	476 (1.92/0.52)	1.70×10^8	1450	333 (1.75/0.06)	1.83×10^8	1.07	1.53	0.99
Decreased	2947	630 (1.57/0.05)	2.34×10^8	2027	333 (1.73/0.04)	5.48×10^7	0.69	1.30	2.94
	2931	404 (1.95/0.04)	7.68×10^8	1367	535 (1.52/1.32)	2.21×10^8	0.47	0.35	1.62
	2412	427 (1.75/0.06)	2.81×10^8	14782	615 (1.92/0.52)	2.22×10^7	6.13	4.26	77.57
	2789	402 (1.73/0.04)	2.06×10^9	8112	786 (1.85/0.03)	1.85×10^8	2.91	1.49	32.39
	11719	182 (1.84/1.69)	3.18×10^9	11761	108 (1.57/0.05)	8.15×10^8	1.00	1.69	3.92
	6236	135 (1.74/0.02)	2.97×10^8	5102	325(1.95/0.04)	4.38×10^8	0.82	0.34	0.55

© 2020 by the authors. Licensee MDPI, Basel, Switzerland. This article is an open access article distributed under the terms and conditions of the Creative Commons Attribution (CC BY) license (http://creativecommons.org/licenses/by/4.0/).
